# Longitudinal Study of Plasma NFL and GFAP as Biomarkers of Alcohol Withdrawal–Associated Brain Injury

**DOI:** 10.1111/adb.70157

**Published:** 2026-05-03

**Authors:** Virgile Clergue‐Duval, Frank Questel, Alexandra Dereux, Elodie Bouaziz‐Amar, Julien Azuar, Romain Icick, Dorian Rollet, Vanessa Bloch, Jérôme Jeanblanc, Cynthia Marie‐Claire, Jean‐Louis Laplanche, Frank Bellivier, Claire Paquet, Mickael Naassila, Florence Vorspan

**Affiliations:** ^1^ Département de Psychiatrie et de Médecine Addictologique APHP, GHU APHP.Nord‐ Université Paris Cité, Site Lariboisière Fernand‐Widal Paris France; ^2^ Université Paris Cité, INSERM, Optimisation thérapeutique en Neuropharmacologie OPTEN U1144 Paris France; ^3^ FHU Network of Research in Substance Use Disorders (NOR‐SUD) Paris France; ^4^ Université Paris Cité, UFR de Médecine Paris France; ^5^ Resalcog ‐ Réseau Pour la Prise en Charge Des Troubles Cognitifs liés à L'alcool Paris France; ^6^ Université Paris Cité, UFR de Pharmacie Paris France; ^7^ Département de Biochimie et Biologie Moléculaire APHP, GHU APHP. Nord ‐ Université Paris Cité, Site Lariboisière Fernand‐Widal Paris France; ^8^ APHP, GHU APHP. Nord ‐ Université Paris Cité, Site Lariboisière Fernand‐Widal, Service de Pharmacie Paris France; ^9^ INSERM UMRS‐1247 Groupe de Recherche Sur L'alcool et Les pharmacodépendances, Université de Picardie Jules Verne Amiens France; ^10^ FHU Améliorer le Pronostic Des Troubles Addictifs et Mentaux Par Une Médecine Personnalisée (A2M2P) Amiens France; ^11^ APHP, GHU APHP. Nord ‐ Université Paris Cité, Site Lariboisière Fernand‐Widal, Centre de Neurologie Cognitive Paris France; ^12^ Université de Picardie Jules Verne, UFR de Pharmacie Amiens France

**Keywords:** alcohol‐related brain damage, alcohol‐related disorder, biomarkers, glial fibrillary acidic protein, hyperglutamatergic, nervous system disorders, neurofilament light chain

## Abstract

Plasma neurofilament light chain (NFL), glial fibrillary acidic protein (GFAP), tau protein and ubiquitin carboxy‐terminal hydrolase L1 (UCHL1) are candidate biomarkers of alcohol withdrawal (AW)‐associated brain toxicity, as they are biomarkers of axonal, neuronal or glial injury. The aim of this study was to investigate the changes of these biomarkers during AW in patients with severe alcohol use disorder (AUD). Plasma NFL, GFAP, tau and UCHL1 levels were measured, with SIMOA, at three times: on Day 1 (T1), on Day 3 or 4 (T2) and on Day 13, 14 or 15 (T3) of AW. They were analysed with a linear mixed model adjusted for age, sex and body mass index. Changes in these levels according to AW symptom severity were evaluated. Twenty‐four inpatients with severe AUD were included: 20 men (83.3%), aged 47.4 years [±11.3], with symptoms requiring a median equivalent‐diazepam dose of 0.81 mg/kg at T1. A significant increase was observed for NFL level from T1 to T2 (β = 0.349, *p* = 0.035), but not for GFAP, tau or UCHL1 levels. In AW symptom severity analyses, a significant positive association was found with equivalent‐diazepam dose required × T1–T2 time interaction factor for NFL (β = 0.161, *p* = 0.028) and for GFAP (β = 0.400, *p* = 9.9 × 10^−4^). This longitudinal study provided preliminary indication that brain injury could occur within the first days of AW, especially in patients with severe pharmacological dependence. Plasma NFL and GFAP are promising biomarkers of AW‐related brain pathology and should be investigated as biomarkers of therapeutic response to test innovative drug strategies for preventing this toxicity.

**Trial Registration:** Clinical Trials: NCT05216705

## Graphical abstract

Brain injury could occur within the first days of alcohol withdrawal, especially in patients with severe pharmacological dependence. Plasma NFL and GFAP are promising biomarkers of alcohol withdrawal‐related brain pathology.

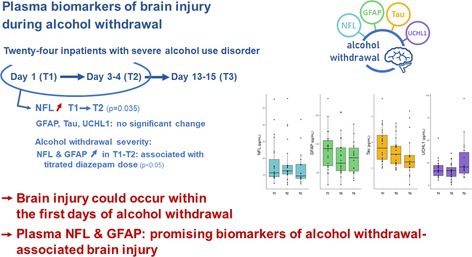

## 1 Introduction

Alcohol use disorder (AUD) is burdensome worldwide, with an estimated lifetime prevalence of 8.6% [1] and high morbidity and mortality [2, 3]. Many AUD patients experience alcohol withdrawal (AW) syndrome, particularly two‐thirds of patients with severe AUD [4]. The progressive course of pharmacological alcohol dependence and AW syndrome onset are related to homeostatic shifts and neuroadaptation to the condition of chronic alcohol intoxication [5]. AW syndrome is a critical factor in AUD patients, as it leads to a high incidence of hospitalization [6]. AW is known to induce epileptic seizures and *delirium tremens* (DT) in the absence of appropriate benzodiazepine treatment [7, 8]. These two major complications are known to be neurotoxic [8, 9]. The evaluation of the risk of the occurrence of these complications, and therefore the management of benzodiazepine therapy, which is the current consensus to prevent them, is guided by the adrenergic and clinical signs observed on clinical examination [7, 9, 10]. Nevertheless, numerous lines of evidence support the hypothesis of brain neurotoxicity even in the absence of epileptic seizures or DT, notably the hyperglutamatergic state or the sometimes associated Wernicke's encephalopathy (WE) [11, 12, 13]. The repetitive episodes of AW in a patient's addictive trajectory could contribute to the development of alcohol‐related brain damage and alcohol‐related cognitive impairment [11], as observed in chronic traumatic encephalopathy [14], The identification of new therapeutic strategies for AW to alleviate AW symptoms and prevent acute and long‐term complications is crucial [7, 13], This identification, in turn, requires the identification of new biomarkers, in particular those that are sensitive to changes during patient management, minimally invasive and accessible in routine clinical practice. In this context, the study of novel plasma biomarkers has emerged as a popular research topic, with neurofilament light chain (NFL), a biomarker of axonal toxicity [15, 16], being the main candidate [17]. Three studies have recently been conducted on the measurement of plasma or blood NFL levels in the context of alcohol cessation in humans [18, 19, 20]. In a first cross‐sectional pilot study, our team reported that plasma NFL levels were higher in severe AUD inpatients beginning AW than in AUD patients who had been alcohol detoxified for more than 3 months, and the plasma NFL level was higher in severe AUD inpatients in AW with neurological signs of WE than in those without these signs [18]. In a cross‐sectional study, Hou et al. reported a higher plasma NFL level in AUD inpatients beginning AW with subsequent DT complications in the following days than in those who did not experience these complications [19]. Huang et al. showed, in the first published prospective study, that blood NFL levels were significantly reduced after 1 week of alcohol cessation in alcohol‐dependent inpatients; however, these researchers did not provide information on AW management or differences in NFL level changes according to the severity of AW symptoms [20]. In one published study, an animal model of AW was evaluated. In rats subjected to the Alcohol Deprivation Effect model, plasma NFL levels increased after AW without drug or vitamin treatment [18]. In addition, we identified three other candidate biomarkers of neurotoxicity previously used as biomarkers of traumatic brain injury or neurodegenerative disease and capturing distinct brain injury phenomena. These proteins include glial fibrillary acidic protein (GFAP), a complex biomarker of both glial injury and astrogliosis; tau protein, a biomarker of neuronal injury; and ubiquitin carboxy‐terminal hydrolase L1 (UCHL1), a biomarker of neuronal injury and proteasome activity [18, 21, 22, 23, 24, 25]. GFAP is a biomarker of interest due to its applications in central nervous system injuries, notably in traumatic brain injury but also as a biomarker related to neuroinflammatory and neurodegenerative diseases [25]. Moreover, astrocytes play a central role in synaptic function, and particularly in the regulation of hyperglutamatergia [26]. In the cross‐sectional study mentioned above, our team observed a positive association between tau and UCHL1 levels and the dose of diazepam required for managing AW symptoms, reflecting AW symptom severity [18]. We hypothesize that brain injury occurs during alcohol withdrawal, particularly in patients with greater pharmacological dependence, and this is reflected in an increase in plasma NFL level, and secondarily in other plasma biomarkers.

The objective of this study was therefore to use a longitudinal design to assess the time course of the levels of four plasma biomarkers of brain damage (NFL, GFAP, tau protein and UCHL1) during medically managed AW, within the first 48–72 h and over 2 weeks, in severe AUD inpatients and to investigate associations with AW symptom severity.

## 2 Materials and Methods

### 2.1 Sample Enrollment

Patients were included at the Addiction Medicine Inpatient Unit of Fernand‐Widal Lariboisière University Hospital, Paris, France, between April 2023 and April 2024. The inclusion criteria were as follows: a diagnosis of severe AUD based on the criteria in the Diagnostic and Statistical Manual of Mental Disorders, fifth edition (DSM‐5); inpatient treatment for medically managed AW; and age from 18 to 65 years. The exclusion criteria were as follows: last alcohol use more than 24 h before admission; use of other substances in the previous month (except tobacco); other substance use disorders (except tobacco); treatment with opioid agonist therapy (methadone or buprenorphine); cirrhosis at the decompensated stage (Child–Pugh Class B or C); liver failure; severe acute alcoholic hepatitis; renal failure; epileptic seizure; stroke or head trauma in the previous 3 months; known neurodegenerative diseases or known severe alcohol‐related cognitive impairment; seropositivity for human immunodeficiency virus, hepatitis C virus or syphilis; pregnancy; and non‐French‐speaking. Alcohol use, the use of other substances (except tobacco) during the study, the occurrence of epileptic seizures and discharge or transfer to another hospital unit ended the patient's participation in this prospective study.

### 2.2 Assessment of Plasma Biomarker Changes

Patient plasma samples were collected three times during inpatient AW: the first day after admission (T1), the third or fourth day (T2) and the thirteenth, fourteenth or fifteenth days (T3). The samples were collected into EDTA‐containing tubes in the morning under fasting conditions [27]. After sampling, the whole‐blood samples were centrifuged. The plasma was aliquoted and stored at −80°C. The levels of NFL, GFAP, tau protein and UCHL1 were measured using a single‐molecule array immunoassay (SIMOA technology) with a Neurology‐4‐Plex B kit from Quanterix (Massachusetts, USA).

### 2.3 Assessment of Clinical Covariables

The severity of AW symptoms was estimated according to the dose of benzodiazepine per kilogram of weight required the first day after admission to manage AW symptoms. We derived equivalent‐diazepam doses, with a diazepam/oxazepam conversion ratio of 10 mg/30 mg [28]. Benzodiazepine titration was left to the judgement of the clinician based on their evaluation of AW symptoms using the Cushman score [29], which was performed as often as needed. The maximum Cushman score achieved was then recorded to quantify the maximum severity of AW experienced by the patients. The benzodiazepine dose has been described as having better specificity and sensitivity than the symptom scale for reporting AW severity and its course [30]. The occurrence of DT, epileptic seizures or other AW complications was recorded until discharge. The presence of the neurological triad signs of WE (ophthalmoplegia (including nystagmus), ataxia or confusion [31]) the first day after admission was recorded after confirming sobriety. The clinical management team was blinded to the results of the biomarker measurements. Sociodemographic variables and data regarding medication, alcohol and tobacco use were also collected.

### 2.4 Statistical Analysis

Linear mixed effect model for repeated measures was performed to investigate the time variation over the 2‐week (T1–T3) and during the first 48 or 72 h of AW (T1–T2). Analyses were performed after natural logarithm transformation to normalize the distribution of plasma biomarker levels and were adjusted for age, sex and body mass index. Time effects and analyses according to the benzodiazepine dose required on the first day after admission (D1) per kilogram of weight and the presence of at least one neurological sign of WE at D1 were conducted. The main judgement criterion was a change in biomarker level at 2 weeks (T1–T3), on the basis of the increase in NFL levels observed in rats in the Alcohol Deprivation Effect model [18]. Post hoc analyses of the severity of AW symptoms were performed with linear mixed model for repeated measures of changes in biomarker levels according to benzodiazepine dose adjusted to the maximum Cushman score achieved or receiving chronic treatment with serotonin reuptake inhibitors to explore the consistency of the main observed results. Complementary comparisons were performed, in linear regression adjusted for age, sex and body mass index, with samples that were previously collected in another study, from outpatients with AUD and more than 3 months of abstinence to serve as control subjects [18]. Spearman correlation coefficients were calculated between the levels of the four plasma biomarkers at different time points.

### 2.5 Ethics

This study was approved by the official French ethics committee on 16 March 2022 (ID‐RCB number 2021‐A02383‐38, CNRIPH number 21.03792.000050) and was preregistered in Clinical Trial (NCT05216705). Patients provided written informed consent.

## 3 Results

### 3.1 Patient Characteristics and Course of AW

Twenty‐four patients for whom two or three plasma samples were obtained were included. The mean age of these patients was 47.4 years [standard deviation (SD) ± 11.3]. There were twenty men (83%) and four women. The median alcohol intake per day in the last month was 215 g [range: 70–660 g, interquartile range (IQR): 175–320 g]. The median AUD duration was 12.5 years (range: 2–45, IQR: 4.75–27.0). Twenty‐one patients (88%) were current tobacco smokers. The patients' characteristics are presented in Table 1.

**TABLE 1:** Patient Characteristics (*n* = 24).

Characteristics	*N* (%)
Sex (male), *n* (%)	20 (83.3%)
Age (years)	
Mean [SD]	47.4 [±11.3]
Minimum − maximum	27–65 years
Body mass index (kg/m^2^)	
Mean [SD]	24.0 [±3.2]
Minimum − maximum	18.6–30.5
Weight (kg)	
Mean [SD]	73.1 [±8.1]
Minimum − maximum	57–86
Education level attained	
Middle or elementary school	6 (25.0%)
High school without baccalaureate or vocational training qualification	5 (20.8%)
Baccalaureate	4 (16.7%)
Graduate degree	9 (37.5%)
Socioprofessional category^a^	
Employees	6 (25.0%)
Manual workers	10 (41.7%)
Intermediate	4 (16.7%)
Higher	4 (16.7%)
Employment status	
Employed	13 (54.2%)
Unemployed	7 (29.2%)
Disabled	3 (12.5%)
Retired	1 (4.2%)
Marital status	
Single, never in a union	9 (37.5%)
In couple (married or common law)	7 (29.2%)
Divorced or separated	8 (33.3%)
Housing	
Personal housing	15 (62.5%)
Family member's housing	4 (16.7%)
Long‐term social shelter	1 (4.2%)
Homeless (short‐term shelter or not)	4 (16.7%)
Duration of AUD (years)	
Median [minimum − maximum]	12.5 [2–45]
1st quartile; 3rd quartile	4.75; 27.0
Reported alcohol intake per day (grams)	
Median [minimum − maximum]	215 [70–660]
1st quartile; 3rd quartile	175; 320
Number of DSM‐5 criteria for alcohol use disorder	
Median [minimum − maximum]	11 [7–11]
1st quartile; 3rd quartile	10; 11
Tobacco smoking status at admission	
Current smoker	21 (87.5%)
Former smoker or non‐smoker	3 (12.5%)
Cigarettes smoked per day at admission (among current smokers; *n* = 20)	
Median [minimum − maximum]	20 [1–35]
1st quartile; 3rd quartile	20; 24.3
Cigarettes smoked per day at T3 (among current smokers; *n* = 15)	
Median [minimum − maximum]	15 [4–35]
1st quartile; 3rd quartile	10; 20
Serotonin reuptake inhibitor use at admission	12 (50.0%)

^a^ Socioprofessional category according to INSEE classification (French National Institute of Statistics and Economic Studies).

The median diazepam‐equivalent dose per kilogram of weight on the first day after admission was 0.81 mg/kg (range: 0.42–2.6 mg/kg, IQR: 0.58 mg/kg–1.2 mg/kg). Nine patients (38%) had a dose higher than 1 mg/kg. Nine patients presented with at least one WE neurological sign on D1, and among them, four suffered from WE. None of the patients experienced DT, seizures, head injury, other concurrent neurologic events or decompensated psychiatric comorbidity during their stay. Half were undergoing chronic treatment with serotonin reuptake inhibitors (Table 1). The maximum Cushman score was achieved on the day of admission for half of the patients and the following morning upon awakening for the other half of patients. The median Cushman score was 8 (range: 1–13, IQR: 4.75–10). Sixteen (67%) patients had moderate withdrawal symptoms (score ≥ 7), and eight (33%) patients had mild symptoms before adequate control with benzodiazepine. Eighteen patients had three biomarker collection time points, and twenty‐four had only the first two. Of the six patients discharged before T3, three had a favourable evolution of their condition and requested discharge, one was discharged against medical advice, and two were transferred to a specialized medical unit for the diagnosis of a medical comorbidity. One sample (obtained at T2) was uninterpretable and could not be analysed.

### 3.2 Plasma Biomarker Assays and Overall Longitudinal Changes in Plasma Biomarker Levels

**FIGURE 1:**
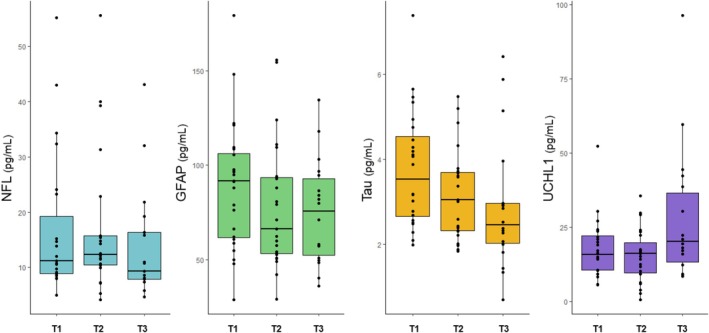
Plasma levels of the four biomarkers—NFL, GFAP, Tau and UCHL1—in 24 inpatients who underwent alcohol withdrawal: the first day after admission (T1), the third or fourth day (T2) and the thirteenth, fourteenth or fifteenth (T3) days.

The plasma levels of the four biomarkers at the three time points are presented in Table 2, Figure 1 and Figure S1. Coefficients of variation for repeated measures of duplicate assays with SIMOA were 3.74% for NFL, 5.47% for GFAP, 5.89% for tau and 6.49% for UCHL1. One outlier subject for the three plasma NFL measurements was excluded from the NFL analyses. In the linear mixed model for repeated measures, a significant increase in plasma NFL level was observed during the first 48 h of AW, between T1 and T2 (β = 0.349, *p* = 0.035), but not between T1 and T3 (β = −0.230, *p* = 0.23) (Table 3). No significant changes were found in plasma GFAP (T1–T2: β = −0.213, *p* = 0.43; T1–T3: β = −0.224, *p* = 0.47), Tau (T1–T2: β = 0.215, *p* = 0.53; T1–T3: β = 0.702, *p* = 0.078) and UHCL1 levels (T1–T2: β = 1.153, *p* = 0.18; T1–T3: β = 0.536, *p* = 0.57) (Table 3). Body mass index was associated with lower plasma NFL (β = −0.100, *p* = 5.5 × 10^−3^) and GFAP levels (β = −0.042, *p* = 0.049), age with higher plasma NFL level (β = 0.024, *p* = 0.015) and the female sex with higher plasma GFAP level (β = 0.349, *p* = 0.049) (Table 3). Spearman correlation coefficients between plasma biomarker levels are presented in Table S1. In complementary comparisons with previously collected measurements in outpatients with AUD and more than 3 months of abstinence [18], significantly higher levels were observed in patients in AW than in abstinent controls for plasma NFL at T1 (β = 0.597, *p* = 5.8 × 10^−3^) and T2 (*p* = 7.8 × 10^−3^) and for plasma GFAP at T1 (*p* = 5.6 × 10^−3^), and not for NFL T3 (β = 0.292, *p* = 0.15), GFAP T2 (β = 0.323, *p* = 0.053) and T3 (β = 0.192, *p* = 0.25) or Tau (*p* > 0.19).

**TABLE 2:** Levels of plasma biomarkers during inpatient alcohol withdrawal on Day 1 (T1), Day 3 or 4 (T2) and Day 13, 14 or 15 (T3) (pg/mL).

		Median	Minimum	1st quartile	3rd quartile	Maximum	*n*
NFL (pg/mL)	T1 (D1)	11.15	5.00	8.89	19.26	55.08	23
	T2 (D3–D4)	12.30	4.17	10.43	15.73	55.56	22
	T3 (D13–D15)	9.31	4.72	7.91	16.33	43.07	17
GFAP (pg/mL)	T1 (D1)	91.55	28.80	61.68	106.29	179.34	24
	T2 (D3–D4)	66.17	29.27	53.14	93.51	155.56	23
	T3 (D13–D15)	75.51	35.98	52.40	92.72	134.47	18
Tau (pg/mL)	T1 (D1)	3.53	1.99	2.65	4.53	7.39	24
	T2 (D3–D4)	3.05	1.83	2.32	3.70	5.48	23
	T3 (D13–D15)	2.45	0.69	2.02	2.96	6.43	18
UCHL1 (pg/mL)	T1 (D1)	15.61	5.59	10.48	22.13	52.22	24
	T2 (D3–D4)	16.06	0.50	9.64	19.83	35.53	23
	T3 (D13–D15)	20.13	8.47	13.16	36.56	96.36	18

Abbreviations: D, day; GFAP, glial fibrillary acidic protein; NFL, neurofilament light chain; T, time point; UCHL1, ubiquitin carboxy‐terminal hydrolase L1.

**TABLE 3:** Linear mixed model for repeated measures of plasma biomarker levels during inpatient alcohol withdrawal, within the first 48 or 72 h (T2) and at 2 weeks (T3), after natural logarithm transformation adjusted for age and body mass index.

	NFL		GFAP		Tau		UCHL1	
	β	*p*	β	*p*	β	*p*	β	*p*
Time T2	0.349	0.035	−0.213	0.43	0.215	0.53	1.153	0.18
Time T3	−0.230	0.23	−0.224	0.47	0.702	0.078	0.536	0.57
Age	0.024	0.015	0.010	0.12	0.001	0.85	0.004	0.74
Sex (female)	0.220	0.42	0.349	0.049	0.268	0.12	−0.231	0.40
Body mass index	−0.100	5.5 × 10^−3^	−0.042	0.049	−0.032	0.12	0.001	0.97
Time T2 × Age	−0.006	0.074	0.002	0.78	−0.008	0.24	−0.030	0.084
Time T3 × Age	0.002	0.54	0.001	0.83	−0.021	0.012	−0.005	0.77

Abbreviations: GFAP, glial fibrillary acidic protein; NFL, neurofilament light chain; T, time point; UCHL1, ubiquitin carboxy–terminal hydrolase L1.

### 3.3 Changes in Plasma Biomarker Levels According to Clinical Variables

With respect to AW symptom severity, during the first 48 h of AW (T1–T2), the equivalent‐diazepam dose required was positively associated with an increase in the plasma NFL level (diazepam dose × time T1–T2 interaction factor: β = 0.161, *p* = 0.028) (Table 4). The plasma GFAP level overall decreased over time T1–T2 (β = −0.513, *p* = 0.043). But the GFAP level change was subject to a significant interaction between diazepam dose and time T1–T2 (β = 0.400, *p* = 9.9 × 10^−4^), In post hoc analyses, additional adjustments according to the maximum Cushman score or taking chronic treatment with serotonin reuptake inhibitors did not affect the positive association between diazepam dose × time T1–T2 interaction factor and the plasma NFL level and plasma GFAP level. With respect to the presence of at least one WE neurological sign at D1, no association was observed with changes in plasma biomarker levels (Table S2).

**TABLE 4:** Linear mixed model for repeated measures of plasma biomarker levels during inpatient alcohol withdrawal within the first 48 or 72 h (T2) and at 2 weeks (T3), according to the dose of diazepam required on D1 per kilogram of weight, after natural logarithm transformation adjusted for age and body mass index.

	NFL		GFAP		Tau		UCHL1	
	β	*p*	β	*p*	β	*p*	β	*p*
Time T2	0.226	0.15	−0.513	0.043	0.154	0.67	1.066	0.23
Time T3	−0.249	0.16	−0.371	0.18	0.721	0.078	0.228	0.81
Diazepam dose	0.309	0.17	−0.308	0.054	0.146	0.37	−0.186	0.55
Age	0.023	0.018	0.011	0.12	−3.6 × 10^−4^	0.96	0.005	0.70
Sex (female)	0.276	0.292	0.331	0.062	0.297	0.076	−0.182	0.50
Body mass index	−0.082	0.019	−0.048	0.035	−0.023	0.28	0.010	0.77
Time T2 × Diazepam dose	0.161	0.028	0.400	9.9 × 10^−4^	0.077	0.64	0.121	0.76
Time T3 × Diazepam dose	−0.072	0.51	0.171	0.33	0.021	0.93	1.016	0.088
Time T2 × Age	−0.007	0.034	−1.5 × 10^−5^	1.0	−0.009	0.23	−0.031	0.074
Time T3 × Age	0.004	0.30	0.006	0.87	−0.021	0.014	−0.017	0.40

Abbreviations: GFAP, glial fibrillary acidic protein; NFL, neurofilament light chain; T, time point; UCHL1, ubiquitin carboxy‐terminal hydrolase L1.

## 4 Discussion

In this observational study on the longitudinal change of four plasma biomarkers during a medically managed AW episode in severe AUD inpatients, we observed an increase in the levels of plasma proteins reflecting a potential particular pattern of brain injury and astrocytic reaction. Plasma NFL level is considered to reflect axonal injury. These changes occurred despite well‐managed medication combining thiamine and benzodiazepines, according to the current state of knowledge, and in the absence of AW complications such as epileptic seizures or DT. The observed increase in plasma NFL levels occurred during the first 3 days of AW. This increase was found, in particular, in patients with the most severe pharmacological dependence, who required higher doses of benzodiazepines and were therefore most exposed to hyperglutamatergia. In a preclinical study, Kahn et al. have shown that glutamate can directly induce the release of NFL [32]. Furthermore, these inpatients had different GFAP changes during the first days, a biomarker of astrocytic activation, astrogliosis and glial injury, but also glial repair [25]. We observed an overall reduction of plasma GFAP level over time, with complex interactions between diazepam dose required and time T1–T2. This result suggests that alcohol cessation reduces glial activation or injury, but to a lesser extent in patients requiring high doses of diazepam to control withdrawal symptoms. These findings on GFAP can be considered in light of the complex associations observed in other central nervous system disorders as biomarkers of acute and/or chronic conditions [25].

This study points to a new way to measure the potential pattern of brain injury that occurs in humans during the first few hours of AW, especially in the most pharmacologically dependent inpatients. It highlights also the relevance of plasma NFL as the most potentially useful plasma biomarkers among the four candidates evaluated. The findings confirmed the increased NFL level observed in rats with the Alcohol Deprivation Effect model and higher levels than abstinent patients [18]. These findings support the time sensitivity of plasma NFL levels during inpatient AW management [20] and suggest that GFAP could also serve as a potential biomarker. These results contrast with those of Huang et al., who reported an overall decrease in plasma NFL levels in AUD inpatients during the first week of alcohol cessation but did not report AW symptoms [20]. This contrast in findings is probably due to greater AW severity in our population than in the Huang et al. population. These findings can be considered in relation to those observed in the cross‐sectional study of Hou et al., which revealed higher plasma NFL levels in inpatients who will develop a DT [19]. Furthermore, we observed that plasma biomarker levels measured 3 days after the beginning of AW might perform better than those measured at admission in assessing or predicting the neurotoxicity of AW and the efficacy of therapeutic drug strategies. This difference in performance could explain the absence of significant results observed with plasma GFAP levels on day one in our first cross‐sectional pilot study [18]. We can also hypothesize that the persistence of high GFAP levels during the early withdrawal period in patients with the most severe pharmacological dependence is due to the astrocytic activation or the sustained presence of glial injury caused by hyperglutamatergia [33], present in the context of heavy chronic alcohol use and persisting into the early withdrawal period [12, 13, 34]. Kahn et al. also found also, in a preclinical model, that the extracellular release of NFL could induce microglial activation [32]. Conversely, plasma total tau protein levels do not appear to be specific to the AW period. However, the plasma tau protein that we were able to detect may lack of brain specificity. Further investigation of the new plasma brain‐derived tau [35, 36] as a biomarker of more severe complications appears justified, especially after the end of the withdrawal period [37]. Moreover, the levels of NFL and GFAP at the three measurement times were highly correlated with each other. There is high interindividual variability, as noted by Bavato et al. in their review of the literature on NFL [17]. This underlines that individual factors other than AW acute brain injury could be associated with these biomarker levels, as observed in cross‐sectional studies investigating alcohol‐induced brain damage [38], alcohol‐related cognitive impairment [39], psychiatric disorders [17] or interaction between substance use disorder and psychiatric comorbidity [40]. In the context of AW, a nutrient‐depleted state could induce different susceptibilities to AW neurotoxicity [12, 41]. Furthermore, measuring their plasma levels at the early days of AW and their changes could serve as new predictive biomarkers for the course of the disorder and guide post‐withdrawal management, including continuation of hospital care for cognitive disorders [42].

Finally, we did not observe a significant association between biomarker levels and the presence of at least one neurological sign of WE in this study. This finding contrasts with that of a previous cross‐sectional study from our group, where NFL, tau and UCHL1 levels were significantly associated with the presence of at least one neurological sign of WE [18]. Several hypotheses could explain these discrepancies, such as the lack of power in the present study, the small number of concomitant neurotoxic events, the fact that WE is a confounding factor in the association observed between biomarker levels and AW symptom severity or the fact that this previous observation of WE symptoms was contingent on other factors. Further studies are needed to elucidate the potential association between neurological signs of WE and biomarkers' plasma levels.

We acknowledge several limitations of our study. We did not include healthy volunteers or controls with AUD who had no history of withdrawal. The absence of a prehospitalization dosage makes it impossible to cover the entire periwithdrawal period. The choice of days for biomarker measurement was based on clinical experience and is questionable. Measuring the levels of these biomarkers at more time points would enable us to determine their accuracy for predicting AW‐associated brain injury events. In addition, the use of the new more specific plasma brain‐derived tau would provide greater brain specificity than the total tau protein [35, 36]. The majority of patients were tobacco smokers, which may be a confounding factor [43]. Furthermore, hydration status was not assessed during the AW, which could have affected plasma concentrations. The study has limited statistical power due to the number of patients included. For instance, the post hoc power estimate for measuring a change between T1 and T2 was 0.70 for GFAP, 0.34 for Tau and 0.39 for UCHL1. Finally, multiple tests were performed, and the relatively small sample size as well as the monocentric design constituted a limitation on the generalization of the finding. They require replication in larger and multicentre study.

The strengths of this study are, first, its innovative scope for AW neurotoxicity assessment with plasma biomarkers and its ability to assess inpatients with severe pharmacological alcohol dependence with a strict exclusion of patients with coaddictions other than tobacco.

## 5 Conclusion

This observational study provided preliminary evidence that brain injury but also repair could occur in the first days of AW, even in the absence of DT or seizures, despite medical management. This injury was notably observed in patients with the most severe pharmacological dependence and involves axonal injury, as indicated by plasma NFL levels. The repair also involved glial cells, as indicated by complex change in plasma GFAP level. Both plasma NFL and GFAP are potentially promising biomarkers of AW‐associated brain toxicity. Nevertheless, it will be crucial to reply and better characterize these results in a larger study. These proteins should also be investigated as biomarkers of therapeutic response in order to test innovative drug strategies for preventing brain injury or accelerating repair during AW.

## Author Contributions

**Virgile Clergue‐Duval:** conceptualization, methodology, investigation, data curation, funding acquisition, formal analysis, project administration, writing – original draft. **Frank Questel:** conceptualization, methodology, resources, writing – review and editing. **Alexandra Dereux:** investigation, writing – review and editing, resources. **Elodie Bouaziz‐Amar:** writing – review and editing. **Julien Azuar:** writing – review and editing, resources. **Romain Icick:** writing – review and editing, resources. **Dorian Rollet:** investigation, writing – review and editing, resources. **Vanessa Bloch:** writing – review and editing, resources. **Jérôme Jeanblanc:** writing – review and editing. **Cynthia Marie‐Claire:** writing – review and editing, resources. **Jean‐Louis Laplanche:** writing – review and editing, resources. **Frank Bellivier:** writing – review and editing, resources. **Claire Paquet:** conceptualization, methodology, writing – review and editing, resources. **Mickael Naassila:** methodology, writing – review and editing, validation. **Florence Vorspan:** conceptualization, methodology, supervision, project administration, validation, funding acquisition, writing – review and editing.

## Funding

This research was supported by the Institut national de la santé et de la recherche médicale (INSERM—French National Institute of Health and Medical Research) with grants from the *INSERM research unit Optimisation thérapeutique en neuropharmacologie U1144* (Université Paris Cité, INSERM) and the Fédération Hospitalo‐Universitaire Network of Research in Substance Use Disorders (Université Paris Cité, INSERM and Assistance Publique—Hopitaux de Paris).

## Ethics Statement

This study was approved by the French ethics committee on 16 March 2022 (ID‐RCB number 2021‐A02383‐38).

## Consent

Patients provided written informed consent.

## Conflicts of Interest

The authors declare no conflicts of interest.

## Supporting information


**Figure S1:** Box plots and individual trajectories of plasma levels of NFL, GFAP, Tau and UCHL1, during inpatient alcohol withdrawal: the first day after admission (T1); the third or fourth day (T2); and the thirteenth, fourteenth or fifteenth (T3) days.
**Table S1:** Spearman's correlation coefficients and *p*‐values between the levels of the four plasma biomarkers at different times.
**Table S2:** Linear mixed model for repeated measures of plasma biomarker levels during inpatient alcohol withdrawal within the first 48 or 72 h (T2) and at 2 weeks (T3), according to the presence of at least one WE neurological sign at D1 (*n* = 9), after natural logarithm transformation adjusted for age, sex and body mass index.

## Data Availability

The datasets presented in this article are not readily available because they are the property of the Assistance Publique—Hôpitaux de Paris and INSERM. Requests to access the datasets should be directed to Pr. Florence Vorspan (florence.vorspan@aphp.fr; ORCID 0000‐0001‐5168‐3727).
