# Supplementary material for: Longitudinal Study of Plasma NFL and GFAP as Biomarkers of Alcohol Withdrawal–Associated Brain Injury
Source: Addict Biol. 2026 May 3;31(5):e70157. doi: 10.1111/adb.70157 (PMC13135891; doi:10.1111/adb.70157)
Supplement: Supplementary file 1 — Figure S1: Box plots and individual trajectories of plasma levels of NFL, GFAP, Tau and UCHL1, during inpatient alcohol withdrawal: the first day after admission (T1); the third or fourth day (T2); and the thirteenth, fourteenth or fifteenth (T3) days. Table S1: Spearman's correlation coefficients and p‐values between the levels of the four plasma biomarkers at different times. Table S2: Linear mixed model for repeated measures of plasma biomarker levels during inpatient alcohol withdrawal within the first 48 or 72 h (T2) and at 2 weeks (T3), according to the presence of at least one WE neurological sign at D1 (n = 9), after natural logarithm transformation adjusted for age, sex and body mass index. [file ADB-31-e70157-s001.pdf]

Supplementary Material

Figure S1. Box plots and individual trajectories of plasma levels of NFL, GFAP, Tau and UCHL1, during inpatient alcohol withdrawal: the first day after admission (T1); the third or fourth day (T2); and the thirteenth, fourteenth or fifteenth (T3) days.

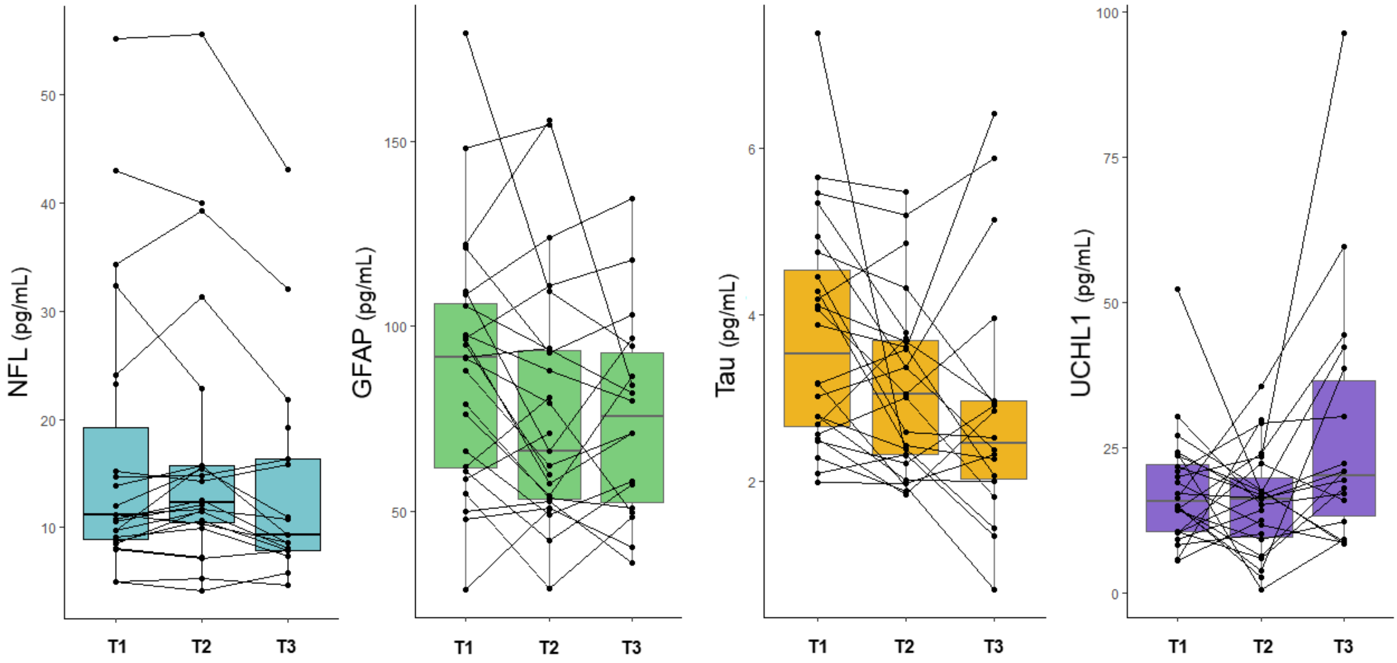

Abbreviations: NFL: neurofilament light chain; GFAP: glial fibrillary acidic protein; UCHL1: ubiquitin carboxy-terminal hydrolase L1; T: time point.

Table S1. Spearman's correlation coefficients and p-values between the levels of the four plasma biomarkers at different times.

	NFL T2	NFL T3	GFAP T1	GFAP T2	GFAP T3	Tau T1	Tau T2	Tau T3	UCHL1 T1	UCHL1 T2	UCHL1 T3
NFL T1	**rho=0.92<br>p=4.0x10^{-6}**	**rho=0.98<br>p=6.1x10^{-6}**	**rho=0.50<br>p=0.016**	**rho=0.45<br>p=0.039**	rho=0.42<br>p=0.098	**rho=0.52<br>p=0.012**	**rho=0.51<br>p=0.017**	rho=0.010<br>p=0.97	rho=0.038<br>p=0.87	rho=0.27<br>p=0.22	rho=0.34<br>p=0.19
NFL T2	NA	**rho=0.93<br>p=2.2x10^{-16}**	rho=0.42<br>p=0.056	**rho=0.50<br>p=0.018**	rho=0.29<br>p=0.27	**rho=0.58<br>p= 5.4x10^{-3}**	**rho=0.63<br>p=2.0x10^{-3}**	rho=0.088<br>p=0.75	rho=-0.055<br>p=0.81	rho=0.35<br>p=0.11	rho=0.49<br>p=0.057
NFL T3		NA	rho=0.40<br>p=0.12	**rho=0.53<br>p=0.038**	rho=0.43<br>p=0.083	rho=0.47<br>p=0.060	**rho=0.61<br>p=0.015**	rho=-0.049<br>p=0.85	rho=0.096<br>p=0.72	rho=0.28<br>p=0.29	rho=0.31<br>p=0.23
GFAP T1			NA	**rho=0.80<br>5.7x10^{-6}**	**rho=0.80<br>9.3x10^{-5}**	**rho=0.59<br>p=3.2x10^{-3}**	rho=0.31<br>p=0.16	rho=0.12<br>p=0.65	rho=0.23<br>p=0.28	rho=0.25<br>p=0.25	rho=0.040<br>p=0.88
GFAP T2				NA	**rho=0.76<br>5.7x10^{-4}**	**rho=0.57<br>p=5.9x10^{-3}**	**rho=0.51<br>p=0.014**	rho=0.23<br>p=0.38	rho=0.0.13<br>p=0.56	rho=0.34<br>p=0.12	rho=022<br>p=0.40
GFAP T3					NA	rho=0.33<br>p=0.18	rho=0.26<br>p=0.31	rho=0.042<br>p=0.87	rho=0.11<br>p=0.66	rho=0.083<br>p=0.75	rho=0.084<br>p=0.74
Tau T1						NA	**rho=0.64<br>p=1.2x10^{-3}**	**rho=0.51<br>p=0.033**	rho=0.022<br>p=0.92	rho=0.13<br>p=0.57	rho=0.19<br>p=0.45
Tau T2							NA	**rho=0.54<br>p=0.028**	rho= -0.29<br>p=0.18	rho=0.37<br>p=0.087	rho=0.44<br>p=0.080
Tau T3								NA	rho= -0.11<br>p=0.67	rho=0.061<br>p=0.82	rho=0.011<br>p=0.97
UCHL1 T1									NA	rho=0.066<br>p=0.76	rho= -0.13<br>p=0.61
UCHL1 T2										NA	**rho=0.51<br>p=0.038**

Abbreviations: NFL: neurofilament light chain; GFAP: glial fibrillary acidic protein; UCHL1: ubiquitin carboxy-terminal hydrolase L1; T: time point; NA: not applicable.

Table S2. Linear mixed model for repeated measures of plasma biomarker levels during inpatient alcohol withdrawal within the first 48 or 72 hours (T2) and at two weeks (T3), according to the presence of at least one WE neurological sign at D1 (n=9), after natural logarithm transformation adjusted for age, sex and body mass index.

	NFL		GFAP		Tau		UCHL1	
	β	p-value	β	p-value	β	p-value	β	p-value
Time T2	0.356	0.032	-0.193	0.47	0.174	0.60	1.058	0.21
Time T3	-0.225	0.24	-0.204	0.51	0.629	0.10	0.435	0.64
WE sign	0.218	0.33	0.088	0.56	-0.164	0.29	-0.242	0.42
Age	0.023	0.018	0.010	0.12	0.002	0.78	0.006	0.69
Sex (female)	0.236	0.38	0.351	0.048	0.281	0.099	-0.211	0.45
Body mass index	-0.093	9.7×10^{-3}	-0.040	0.066	-0.035	0.098	-1.8×10^{-4}	1.00
Time T2 x WE sign	-0.045	0.56	-0.077	0.54	0.152	0.33	0.381	0.33
Time T3 x WE sign	-0.035	0.68	-0.081	0.55	0.330	0.053	0.450	0.28
Time T2 x Age	-0.006	0.084	0.002	0.75	-0.009	0.20	-0.032	0.069
Time T3 x Age	0.002	0.51	0.002	0.79	-0.022	5.8×10^{-3}	-0.007	0.70

Abbreviations: GFAP: glial fibrillary acidic protein; NFL: neurofilament light chain; T: time point; UCHL1: ubiquitin carboxy-terminal hydrolase L1.
